# Immune Influencers in Action: Metabolites and Enzymes of the Tryptophan-Kynurenine Metabolic Pathway

**DOI:** 10.3390/biomedicines9070734

**Published:** 2021-06-25

**Authors:** Masaru Tanaka, Fanni Tóth, Helga Polyák, Ágnes Szabó, Yvette Mándi, László Vécsei

**Affiliations:** 1 MTA-SZTE—Neuroscience Research Group, H-6725 Szeged, Hungary; tanaka.masaru.1@med.u-szeged.hu (M.T.); toth.fanni@med.u-szeged.hu (F.T.); 2 Interdisciplinary Excellence Centre, Department of Neurology, Faculty of Medicine, University of Szeged, H-6725 Szeged, Hungary; polyak.helga@med.u-szeged.hu (H.P.); szabo.agnes.4@med.u-szeged.hu (Á.S.); 3 Department of Medical Microbiology and Immunology, Faculty of Medicine, University of Szeged, H-6720 Szeged, Hungary; mandi.yvette@med.u-szeged.hu

**Keywords:** chronic inflammation, low-grade inflammation, immune tolerance, inflammatory factor, kynurenine, kynurenic acid, depression, bipolar disorder, substance use disorder, post-traumatic stress disorder, schizophrenia, autism spectrum disorder

## Abstract

The tryptophan (TRP)-kynurenine (KYN) metabolic pathway is a main player of TRP metabolism through which more than 95% of TRP is catabolized. The pathway is activated by acute and chronic immune responses leading to a wide range of illnesses including cancer, immune diseases, neurodegenerative diseases and psychiatric disorders. The presence of positive feedback loops facilitates amplifying the immune responses vice versa. The TRP-KYN pathway synthesizes multifarious metabolites including oxidants, antioxidants, neurotoxins, neuroprotectants and immunomodulators. The immunomodulators are known to facilitate the immune system towards a tolerogenic state, resulting in chronic low-grade inflammation (LGI) that is commonly present in obesity, poor nutrition, exposer to chemicals or allergens, prodromal stage of various illnesses and chronic diseases. KYN, kynurenic acid, xanthurenic acid and cinnabarinic acid are aryl hydrocarbon receptor ligands that serve as immunomodulators. Furthermore, TRP-KYN pathway enzymes are known to be activated by the stress hormone cortisol and inflammatory cytokines, and genotypic variants were observed to contribute to inflammation and thus various diseases. The tryptophan 2,3-dioxygenase, the indoleamine 2,3-dioxygenases and the kynurenine-3-monooxygenase are main enzymes in the pathway. This review article discusses the TRP-KYN pathway with special emphasis on its interaction with the immune system and the tolerogenic shift towards chronic LGI and overviews the major symptoms, pro- and anti-inflammatory cytokines and toxic and protective KYNs to explore the linkage between chronic LGI, KYNs, and major psychiatric disorders, including depressive disorder, bipolar disorder, substance use disorder, post-traumatic stress disorder, schizophrenia and autism spectrum disorder.

## 1. Introduction

**Figure 1:**
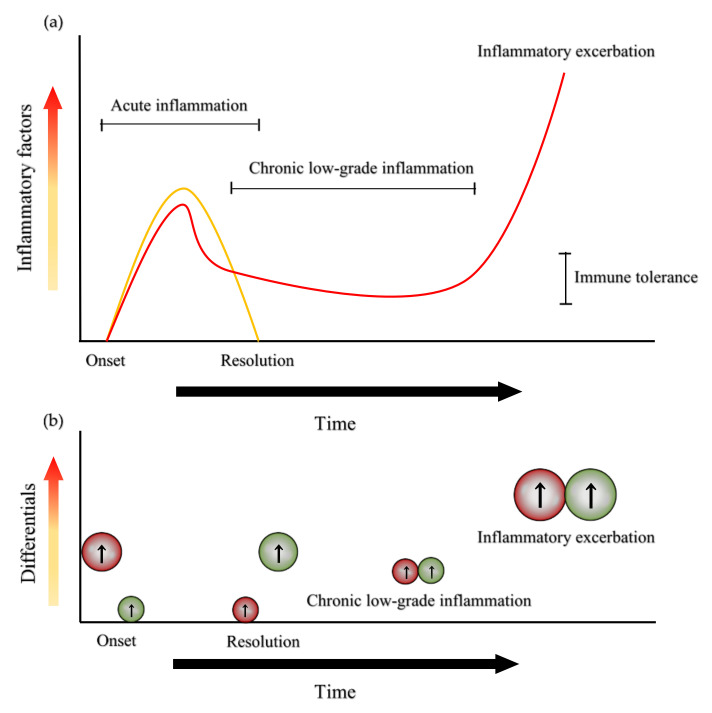
Acute inflammation, chronic low-grade inflammation and the status of pro-inflammatory and anti-inflammatory factors. (**a**) Acute inflammation onsets with the increase of inflammatory factors and is resolved with the decrease of inflammatory cytokines (yellow line). Chronic low-grade inflammation (LGI) is characterized by a period of relatively low-level inflammatory factors that induce immune tolerance. Eventually, inflammatory factors increase, leading to inflammatory exacerbation and contributing to the pathogenesis of various diseases (red line). (**b**) Relative levels of pro-inflammatory (in red) and anti-inflammatory (in green) factors after the onset and before the resolution of acute inflammation, during LGI and at inflammatory exacerbation.

Chronic low-grade inflammation (LGI) has been linked to the prodromal stage of a broad range of chronic illnesses such as cardiovascular-, metabolic-, immunologic-, neurodegenerative- and psychiatric diseases [1]. Chronic LGI is characterized by the long-term of unresolved inflammatory condition in which proinflammatory and anti-inflammatory factors are continuously released and fail to cease their actions (Figure 1a). The long-lasting release of the inflammatory factors initiates compensatory immune suppression and consequently causes immune tolerance, a condition in which the immune system is unresponsive to particular antigens that normally elicit an immune response (Figure 1b).

C-reactive protein (CRP) is a well-established biomarker of inflammation and in LGI the plasma concentration of CRP is between 3 mg/L–10 mg/L. LGI was found to be negatively associated with self-rated physical health score in Health-Related Quality of Life (HRQL) in healthy individuals, but not associated with mental health score in HRQL [2]. Thus, an additional biomarker is necessary to seek the linkage between LGI and mental illnesses. Furthermore, the central nervous system (CNS) is an immune-privileged site that may become the source of inflammatory factors to maintain the immune tolerance. The privileged status of the CNS immune system depends on the integrity of the blood-brain barrier (BBB) and the subsequent influence of inflammatory factors from other parts of the body such as muscles and gut microbiota. The long-term immune tolerance proceeds to significantly higher secretion of inflammatory factors which is linked to inflammatory exacerbation and consequently, pathogenesis of a wide range of neurologic- and psychiatric diseases [3] (Figure 1b).

The tryptophan (TRP)-kynurenine (KYN) metabolic pathway is gaining growing attention as the immune regulator which plays a crucial role of the pathogenesis of a wide range of diseases from cancer to psychiatric disorders. The pathway is a major branch of L-TRP metabolism that is significantly activated during inflammation reaction. Over 95% of L-TRP transforms into a palette of small bioactive molecules with oxidant, antioxidant, neurotoxic, neuroprotective and/or immunomodulatory property. Particularly, KYN, kynurenic acid (KYNA), xanthurenic acid (XA) and cinnabarinic acid (CA) are ligands of the aryl hydrocarbon receptor (AhR) which plays a crucial role in the modulation of inflammation and the subsequent resolution, and the induction of immune tolerance [4].

AhR is a transcription factor that regulates gene expression, but it modulates inflammation through genomic and non-genomic pathways [5]. KYNs are proposed to be emerging players in immunoregulatory networks through AhRs and can be targets for immunotherapy [6]. Strong skeletal muscle mass plays an important role in maintaining effective immune functions. Physical exercise influences the muscle TRP-KYN metabolism and thus energy homeostasis. Intervention through physical exercise program is under thorough study for obesity and chronic illnesses including diseases affecting the CNS [7,8,9].

The activation of the TRP-KYN pathway reversed the progression of the experimental autoimmune encephalomyelitis mouse disease, an animal model of multiple sclerosis (MS) [10]. Monitoring the status of reduction-oxidation as well as KYNs was proposed for useful biomarkers in MS [11]. Cognitive performance, the activation of immune system, and the TRP-KYN pathway were linked in the elderly as well as neurodegenerative diseases [12,13]. KYNA was reported to possess antidepressant-like effects and KYNA analogues were described as potential anti-dementia drugs [14,15]. The TRP-KYN pathway is involved in migraine headache and is one of the potential targets as anti-migraine drugs [16]. Modulation of the pathway was found to reduce or prevent substance abuse [17]. Furthermore, AhR also participates in the gut-brain axis. A novel link between gut microbiota and schizophrenia (SCZ) has been explored in the KYN system [18]. The commensal microflora and microbial AhR agonists are under extensive research in search of the gut microflora-produced biomolecules in influence on CNS and thus novel opportunities for AhR-targeted medications [19]. This review article discusses the metabolites and enzymes of the TRP-KYN metabolic pathway with a special emphasis on its interaction with the immune system, and the major symptoms, pro- and anti-inflammatory cytokines and toxic and protective KYNs of major psychiatric disorders.

## 2. The Kynurenine System

The essential amino acid TRP is metabolized into several different bioactive molecules such as nicotinamide adenine dinucleotide (NAD^+^), serotonin (5-hydroxytryptamine, 5-HT), and melatonin (MT). Only 1–5% of TRP is utilized through the methoxyindole pathway, synthesizing 5-HT and MT; however, 90–95% of TRP is utilized in the TRP-KYN metabolic pathway which is leading to the synthesis of NAD^+^ and other bioactive molecules. Many cell types and organs have important roles in the pathway, such as the brain, liver, intestine and immune cells. NAD^+^ is a main cofactor of the electron transport in the mitochondrial respiratory chain, which is essential for the synthesis of adenosine triphosphate (ATP), ATP also plays an important role in the brain’s glycogen storage and in several enzyme reactions. In addition to NAD^+^, several bioactive molecules are synthesized in the pathway [20].

**Figure 2:**
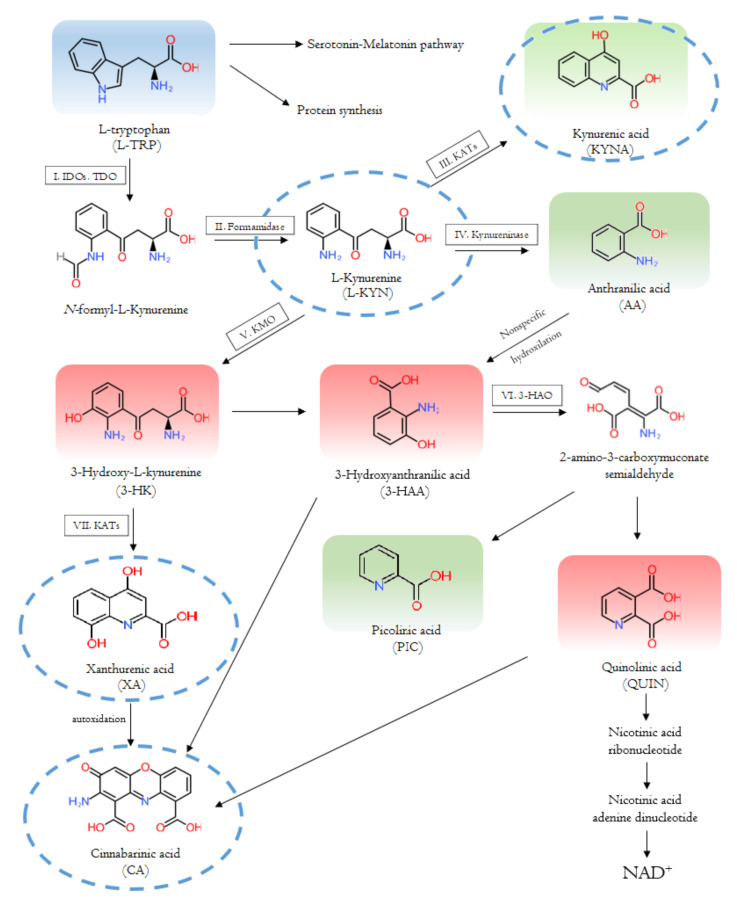
The tryptophan-kynurenine metabolic pathway and bioactive kynurenine metabolites. The pathway depends on the type of cells. In some cells some enzyme is missing, and thus, some metabolite is not produced. The kynurenine metabolites are multifarious molecules with various properties. The aryl hydrocarbon receptor (AhR) ligands are circled with a blue dotted line; toxic kynurenines (KYNs) are in red shade, and protective KYNs are in green shade [20].

Firstly, L-TRP is converted to N-formyl-kynurenine by tryptophan 2,3-dioxygenase (TDO) or indoleamine 2,3-dioxygenase (IDO) 1 or 2. N-formyl-kynurenine is degraded to L-KYN by formamidase. L-KYN is metabolized by three ways: to KYNA by kynurenine aminotransferase (KATs), to 3-hydroxy-kynurenine (3-HK) by kynurenine 3-monooxygenase (KMO) and to anthranilic acid (AA) by kynureninase. KAT enzymes also transform 3-HA to XA. 3-HK and AA are transformed to 3-hydroxyanthranilic acid (3-HAA) by kynureninase or in a non-specific hydroxylation, respectively. In this case, 3-HAA is converted to picolinic acid (PIC) by 2-amino-3-carboxymuconate-semialdehyde decarboxylase, and to quinolinic acid (QUIN) by 3-hydroxyanthranilate dioxygenase. XA is converted to CA by autoxidation. CA is also produced from 3-HK or QUIN. Eventually, QUIN is transformed into nicotinic acid ribonucleotide, then to nicotinic acid, leading to the formation of NAD^+^ [20] (Figure 2).

### 2.1. Enzymes and Metabolites of the Tryptophan-Kynurenine Pathway

Many studies have reported association between the TRP-KYN metabolic pathway, its metabolites and enzymes and the immune systems. TRP degradation via the pathway is activated by an acute immune response in a wide range of diseases, including immune disorders, neurodegenerative and psychiatric diseases, as well as cancer [21,22,23,24,25]. The enzymes involved in the pathway play a major role in various immunological and inflammatory processes. Furthermore, the metabolites of the pathway are also involved in numerous pathological and physiological processes [13,25]. These metabolites can be neuroprotective, neurotoxic, oxidant, antioxidant and/or immune modifiers [26]. In addition, KYN metabolites have proinflammatory, anti-inflammatory and immunosuppressive properties [27]. They regulate the proliferation and function of several immune cells [28]. The pathway is important in mediating the equilibrium between activation and inhibition of the immune system. It is a controller of innate and adaptive immune responses [29].

#### 2.1.1. The Interaction with the Immune System

The interaction of the TRP-KYN metabolic pathway with the immune system were demonstrated in viral or bacterial inflammatory processes [30], including in autoimmune diseases such as systemic lupus erythematosus (SLE) and Sjögren’s syndrome (SjS). Analysis of peripheral blood of SjS patients found higher expression of IDO-1 in the patients’ dendritic cells, compared to the dendritic cells of healthy controls [31] (Table 1). In SjS patients, high levels of IDO were reported in T cells and in antigen-presenting cells (APC), compared to healthy controls [31,32]. In SLE patients’ plasma and cerebrospinal fluid (CSF), lower TRP levels and elevated QUIN levels were observed compared to healthy controls [33]. There was a significant positive correlation between the KYN/TRP ratio and tumor necrosis factor (TNF)-α levels, indicating a link between the pro-inflammatory pathway and KYNs [33] (Table 1).

KYNs are key factors in T cell mediated immune responses. KYN can inhibit antigen-specific T cell proliferation and cause apoptosis [29]. KYNs lead to cell death in the T helper type 1 (Th1) cells, at the same time they upregulate T helper type 2 (Th2) cells. This switches the balance between the Th1–Th2 ratio to Th2 [29,51]. Elevation of the TNF-stimulated gene 6 (TSG-6) expression by KYNA and novel KYNA analogues may be one of the mechanisms responsible for their suppressive effect on TNF-α production [52]. Targeting the metabolic pathway with the purpose of repairing the imbalances of KYN metabolites can be a potential approach to improve symptoms.

#### 2.1.2. Tryptophan 2,3-Dioxygenase

**Figure 3:**
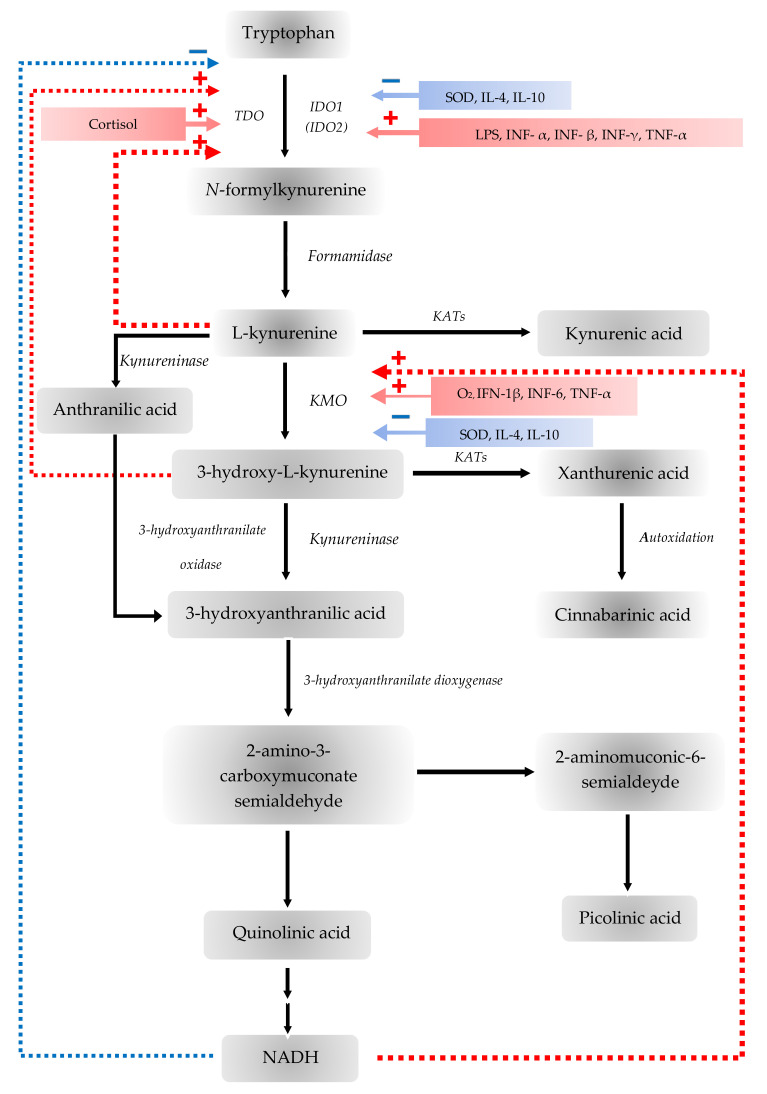
Stimulators (red) and inhibitors (blue) of the tryptophan-kynurenine metabolic pathway. The tryptophan 2,3-dioxygenase (TDO) is stimulated by cortisol and 3-hydroxyanthranilic acid (3-HA) but inhibited by nicotinamide adenine dinucleotide (NADH) forming a negative feedback loop. The indoleamine 2,3-dioxygenases (IDOs) are stimulated by interferon (IFN)-β, IFN-γ, tumor necrosis factor (TNF)-α and lipopolysaccharide (LPS) but inhibited by IL-4, IL-10 and superoxide dismutase (SOD). Kynurenine 3-monooxygenase (KMO) is stimulated by IFN-1 β, IFN-6, TNF-α, oxygen molecule (O_2_) but inhibited by IL-4, IL-10 and SOD. KMO is also inhibited by NADH forming another negative feedback loop.

The TDO enzyme is mainly expressed in liver tissue and is responsible for the breakdown of TRP [36,53]. The TDO enzyme also occurs in the brain. Two variants were identified and expressed at different levels in the brain of mouse, and it was proposed that its role is crucial in postnatal development [54,55]. Furthermore, the TDO-AhR pathway is activated and related to the malignant progression and low survival in brain tumors. The TDO-induced KYN represses the body’s anti-tumor immune response, thereby promoting tumor cells motility and survival through the AhR system [34]. During inflammation of the microenvironment and tumor progression, KYN is pronounced in adequate amounts to activate AhR in humans [34] (Table 1). In addition, an increased TDO expression was described in many tumor types, for example, breast cancer, melanoma or lung cancer [35,36] (Table 1). The main determinant of TRP availability is the TDO enzyme, the expression of TDO can be stimulated by estrogens, corticosteroids, heme, as well as TRP itself [35,56,57]. NADH and NADPH inhibit TDO expression through a negative feedback loop [54] (Table 1, Figure 3).

#### 2.1.3. Indoleamine 2,3-Dioxygenase

IDOs are responsible for the first step in extrahepatic TRP metabolism. IDOs are expressed by APCs, vascular endothelium, epithelial - and tumor cells [58]. This is a widely inducible enzyme, which occurs in most tissues in human [36,59]. IFN-γ-susceptible elements regulate the IDO gene that bind to activate signal transducer and activator of transcription 1, nuclear factor-κB and IFN regulatory factor-1 [60]. However, IDO can be most strongly induced by IFN-γ, as well as many different inflammatory cytokines and mediators, while nitric oxide or the predominance of TRP inhibits these enzymes [54,61]. The IDO induction is inhibited by certain anti-inflammatory cytokines and is potentiated by pro-inflammatory cytokines, such as TNF-α [54]. Consequently, it is hypothesized that the state of the IDO enzymes, is affected by the balance between pro- and anti-inflammatory cytokines [54]. Guillemin et al. have described that several brain cells, including astrocytes, microglial cells, endothelial cells or the human neurons expressed the IDO enzymes [62]. The effect of IDOs on the immune system was first described in the prevention of T-cell-mediated fetal rejection, when it was demonstrated that IDO enzymes synthesized in placental cells protect the fetus from maternal T cell-mediated attack [63]. Furthermore, studies described that IDO is expressed in several tissue types, such as the human lung, small intestine, over and above the placenta already mentioned, and it is upregulated during inflammatory processes, since the IDO enzymes have a physiologically key role in regulating the immune response to antigenic challenges on the surface of the gastrointestinal mucosa [58,64]. The majority of tumor cells overexpress IDOs, either in tumor cells themselves or in tumor-associated cells such as macrophages, dendritic cells or endothelial cells [37]. Due to the inducible nature of the IDO enzymes, it is expressed in the tumor after a certain degree of inflammation which, in turn, activate the tumor. Expression of IDOs is induced by T-cell activation or inflammation which is subsequently suppressed [37]. This is beneficial if the IDO controls harmful inflammation or creates APC tolerance to apoptotic cells, but it is extremely harmful if it suppresses the immune response against the tumor [37] (Table 1, Figure 3). The elevated expression of IDOs is an unfavorable prognostic factor in ovarian cancer, melanoma, breast cancer, brain tumors, glioma and colon cancer, among others [35,37]. The upregulation of IDOs is associated with increased T cell infiltration and inflammation, which indicates anti-tumor immune response and favorable prognosis [37]. In addition, a number of studies presented altered IDO expression in various neurological diseases. An elevated IDO enzyme activity has been described in cerebral ischemia, Parkinson’s disease (PD), Huntington’s disease (HD) and other neurodegenerative diseases, among others [25] (Table 1). The activation of IDO results in a complex immunomodulatory effect which is considered to be essential for the development of both physiological and pathological immune tolerance. Furthermore, the parallel occurrence of autoimmune and immunological process can be a self-defense response of the body [25].

Natural killer (NK) cells take part in innate immunity, destroying pathogens and transformed cells. IDO metabolites can suppress NK cell proliferation and function [65]. KYN has a pro-apoptotic effect on NK cells, which is mediated by ROS [66]. IDO can regulate invariant natural killer (iNKT) cell response and KYN, 3-HK and 3-HAA switch the cytokine balance to Th2, decreasing interferon (IFN)-γ [67]. IDO inhibition shifts the cytokine response to Th1 with a decrease in interleukin (IL)-4.

#### 2.1.4. Kynurenine Aminotransferases

KATs catalyze the conversion L-KYN to KYNA. In the human brain there are four isoforms of KATs, including KAT I, glutamine transaminase K/cysteine conjugate beta-lyase (CCBL) 1, KAT II, aminoadipate aminotransferase, KAT III, glutamine transaminase L/cysteine conjugate beta-lyase CCBL 2 and KAT IV, glutamic-oxaloacetic transaminase 2/mitochondrial aspartate aminotransferase [68]. The isoform liable for KYNA synthesis is the KAT II in human brain [68,69]. These enzymes have a broad substrate specificity and function, being biologically active as homodimer with pyridoxal 5’-phosphate-dependent enzyme family [68,70,71]. In addition to the irreversible transamination of L-KYN to KYNA, KATs can catalyze the conversion of 3-HK to XA [70]. The permeability of KYNA through the BBB is low [72]. KATs activity occurs in astrocyte cells [69,73]. KYNA targets on a number of sites in the CNS and participates in several neurodegenerative diseases involving cognitive impairment [71,74,75,76]. KYNA alters cytokine release from human iNKT cells via G-protein coupled receptor 35 (GPR35) [77]. The increased levels of plasma KYNs was observed in disorders with iNKT cell involvement such as autoimmune diseases and multiple sclerosis [78,79]. The KYNA-centric hypothesis holds that KYNA is well capable of modulating neuropathological conditions [80]. Therefore, isoenzymes involved in the formation of KYNA are considered to be potential targets in the regulation of cerebral KYNA [68,81,82,83,84].

Changes in KYNA concentrations have been described in several different neurological disorders, including MS, PD, Alzheimer’s disease, HD, epilepsy, amyotrophic lateral sclerosis, as well as SCZ [25,39,40,41,42,43,44,45,46] (Table 1). The inhibition of TNF-α can be a potential treatment for various inflammatory diseases [85]. KYNA suppresses the production of TNF-α in mononuclear cells and in CD14^+^ peripheral blood monocytes. KYNA inhibits TNF-α at a transcriptional level [86]. An elevated brain TNF-α level plays a pivotal role in the pathogenesis of neurodegenerative disorders [87]. KYNA inhibition of TNF-α production may consequently be a significant factor in neuroprotection.

Compared to healthy controls SCZ patients have higher KYNA levels in the CSF and in CNS regions [48] (Table 1). In SCZ the Th1 response is in part inhibited, whilst the Th2 response is over-activated leading to a Th1/Th2 imbalance, which is linked to astrocyte activation [88]. Astrocytes can generate vast amounts of KYN and KYNA [62]. The functional abundance of astrocytes can result in additional KYNA accumulation. Since astrocytes lack KYN hydroxylase, this leads to KYNA accumulation in the CNS. Consequently, immune-mediated disturbance of glutamatergic-dopaminergic neurotransmission can bring about the clinical symptoms of SCZ [47] (Table 1).

There is a link between TRP, KYN and KYNA peripheral blood levels and depressive symptoms during IFN-α treatment [89]. Van Gool et al. also found a connection between IFN-α immunotherapy and psychiatric side effect [90]. IFN-α induces IDO, which converts TRP to KYN leading to a shortage of 5-HT, which may result in depression [38,91] (Table 1). IDO induction, a switch toward the TRP-KYN metabolic pathway and the disruption of the equilibrium between neurotoxic and neuroprotective actions all confirm the significance of KYN system activation in depression [92]. Psychological stress elevates brain KYN levels and switches TRP metabolism to the TRP-KYN metabolic pathway [14,93].

#### 2.1.5. Kynurenine 3-Monooxygenase

KMO is responsible for the conversion of L-KYN to 3-HK. It is a mitochondrial flavoprotein, using nicotinamide adenosine dinucleotide phosphate (NADPH) and O_2_ for the catalyzed reaction [94]. In various neurodegenerative diseases an elevated 3-HK level has been observed, as an endogenous oxidative stress generator [39,95]. An elevated KYNA level has been observed in the CSF of patients with SCZ, as KMO is responsible for the conversion of L-KYN to 3-HK, based on the fact that the amount of L-KYN available for KYNA synthesis decreases; thus, the increase in KYNA in SCZ is due to increased KYN and overexpression of KAT, irrespective of changes in KMO [96]. L-KYN has been reported to support the regulatory T-cells and tumor formation through the AhR as well as the activation of the adenylate- and guanylate-cyclase pathways [27,97,98]. Presumably, an increase in kynurenine metabolism via over-expression of KMO may provide a protection against tumorigenesis [27]. Nevertheless, upregulation of KMO can cause the formation of other neuroactive molecules, such as QUIN, HAA and 3-HK which have a ROS-generating effect [27]. Inhibition of KMO, in turn, enhances the formation of neuroprotective KYNA, which plays an important role in immunomodulation [27,99,100,101]. The KMO expression is regulated in lipopolysaccharide-induced systemic inflammation along with a significant increase in pro-inflammatory cytokines in the CNS of rats [102]. Furthermore, KMO activity has been studied in bipolar disorder (BD), a reduced KMO gene expression has been described in the prefrontal cortex of patients with BD with psychotic feature compared with BP patients without psychotic traits [49] (Table 1).

KMO expression and activity have been studied in autoimmune diseases. The link to the immune system appears to exist via AhRs which play a key role in regulating the differentiation of pro-inflammatory Th17 cells [50]. Increasing KYN metabolism through KMO can be protective against tumor formation. Nevertheless, KMO upregulation also produces the following metabolites 3-HK and QUIN with reactive oxygen species (ROS) generating properties and neurotoxic effects [103]. T helper type 17 (Th17) cells express the enzyme KMO. Addition of exogenous KYN or inhibition of KMO activity caused an amelioration in Th17 lineage differentiation in a mouse model of autoimmune gastritis [50]. KMO facilitates KYN metabolism, decreasing KYN levels and subsequently reducing Th17 cell formation and IL-17 production. Thus, KMO inhibition intensified inflammation through the formation of Th17 cells [50].

The inhibition of KMO elevates KYNA production. KYNA has immunomodulatory effects via GPR35 receptors and AHRs [101]. GPR35 is highly expressed in human CD14^+^ monocytes, T cells, neutrophils and dendritic cells, while it is expressed in lower levels in B cells, eosinophils, basophils and iNKT cells [77,104]. KYNA attenuates inflammation by restricting TNF production in macrophages via GPR35 receptors [104]. In KMO^−/−^ mice serum, lower cytokine and chemokine levels, whilst higher KYN and KYNA levels were found compared to the wild type [105]. Elevated levels of KYN and KYNA are the vital components in the decreased inflammatory responses found in KMO^−/−^ animals.

## 3. Symptoms, Inflammatory Status and Kynurenines in Psychiatric Disorders

This section overviews and discusses the major symptoms, inflammatory status and the kynurenine system of psychiatric disorders in clinical human studies. The reference priority is given to the following order: meta-analysis, systematic review, case-control study and expert review. Depression, anxiety and cognitive impairment are the most common symptoms of psychiatric disorders that may concur and/or sway during progression and comorbidity frequently occurs in mental illnesses, which renders the exact diagnosis even more difficult. Scrupulous studies are underway to untangle the thread of pathophysiology of mental disorders and their comorbidities not only in clinical medicine, but also in animal studies [106,107,108,109]. Psychological stress, especially depression has been found to be a risk factor for dementia, a prognostic biomarker for stokes and a therapeutic target for meaning-centered psychotherapy in depression, and animal-assisted and pet-robot interventions in dementia [110,111,112,113]. Four main representative psychiatric symptoms including positive, negative and cognitive symptoms, and anxiety are reviewed. Psychiatric disorders present inflammatory signs in serum, CSF and/or the brain tissue samples in which pro-inflammatory and anti-inflammatory cytokine levels can be detected and measured. The simultaneous alternations of KYN metabolism take place under inflammation, disturbing a balance of toxic and protective KYN metabolites. 

### 3.1. Major Depressive Disorder

MDD is a mental disorder with at least two weeks of low mood, often accompanied by low self-esteem, loss of interest, low energy and pain without a cause. Less than one-fifth of MDD patients experiences positive psychotic symptoms such as either delusions, hallucinations or both [114]. The mean score of the Hamilton Rating Scale for Depression, Scale for the Assessment of Negative Symptoms (SANS) and negative symptom scale of Positive and Negative Symptom Scale of the patients with MDD were significantly higher than those of control subjects, validating the clinical significance of negative symptoms and depressive symptoms in MDD patients [115]. Cognitive impairment in patients with MDD is often overlooked and may precede after symptoms of MDD, such as sleep, appetite and affective symptoms [116]. Generalized anxiety disorder (GAD) often co-occur in MDD. Many symptoms overlap with MDD and GAD, such as irritability, restlessness, sleep problems and concentration difficulty [117] (Table 2).

Regarding inflammatory cytokines, meta-analyses reported strong evidence of significantly increased levels of CRP, IL-1, IL-6, TNF-α and sIL-2R in serum of MDD patients [118,119,120,121,122,123]. Decreased levels of CRP and IL-6 were observed after antidepressant treatment [124]. Higher concentration of CCL2/MCP-1 was also reported in MDD patients. CSF levels of IL-6 and IL-8 were significantly increased in patients with MDD [125] (Table 2).

Regarding the KYN system, meta-analyses reported the decreased levels of plasma TRP, KYN and KYNA in patients with MDD, and the increased level of QUIN was observed in antidepressant-free patients. The increased QUIN immunoreactivity was detected in the prefrontal cortex and hippocampus of the postmortem brain tissues from patients with MDD [126,127]. Magnetic resonance spectroscopy showed a higher turnover of cells with KYN and the 3-HAA/KYN ratio in adolescent depression. The findings are in accordance with the activation of the TRP-KYN pathway by pro-inflammatory cytokines activating IDO, and KMO enzymes toward 3-HK and QUIN branches, leading to higher levels of toxic 3-HK and QUIN [128] (Table 2).

### 3.2. Bipolar Disorder

BD is a mental disorder that causes alternating periods of depression and mania. Positive symptoms regularly occur to BD with prevalence rates ranging from 20 to 50% in acute bipolar mania [129]. Cognitive impairment is present in the minority of BD patients. SANS, Brief Psychiatric Rating Scale and Social and Occupational Functioning Assessment Scale showed that negative symptoms were present in more than a quarter of the patients. The patients had more sever affective flattening, alogia, anhedonia-asociality and avolition-apathy [130]. Cognitive deficits of verbal and visual memory, and executive tasks have been demonstrated during depressive episodes, while executive dysfunction and attention deficits have been reported during manic episodes [131,132]. Many patients with BD experience at least one anxiety attack [133] (Table 2).

Regarding inflammatory cytokines, four meta-analyses of serum or plasma samples from BD patients invariably reported significantly increased levels of TNF-α and sIL-2R; IL-4, IL-6, IL-1RA, sIL-6R and TNFR1 levels were significantly increased in two meta-analyses; IL-10 levels were significantly increased in one meta-analysis [123,134,135,136]. A meta-analysis of CSF samples from BD patients reported increased IL-1β levels [125] (Table 2).

Regarding the KYN system a case-control study showed that KYNA levels were reduced and the 3-HK/KYN and 3-HK/KYNA ratio was increased in BD compared to healthy control [137]. However, a meta-analysis reported no significant difference of TRP and KYN levels, KYN/TRP and KYNA/QUIN ratios in serum from BD patients [138] KYNA was significantly increased in CSF of BD patients [124] (Table 2).

### 3.3. Generalized Anxiety Disorder

GAD is a mental disorder characterized by excessive, uncontrollable and irrational anxiety. GAD is associated with the severity of positive symptoms such as delusions and hallucinations [139]. More than quarter of GAD patients showed negative symptoms [140]. Patients with GAD have an impaired cognitive function, particularly in attention and working memory [141] (Table 2).

On the status of inflammatory cytokines, CRP of blood, serum or plasma samples was significantly raised in GAD by meta-analysis, and IFN-γ and TNF-α levels were significantly increased in GAD in at least two or more studies [142]. Lower levels of IL-10 and higher ratios of TNF-α/IL10, TNF-α/IL4, IFN-γ/IL10 and IFN-γ/IL4 were observed in the serum of GAD patients, showing significantly increased pro- to anti-inflammatory cytokine ratios, which suggests a distinct cytokine imbalance [143] (Table 2). 

On the KYN system, the plasma KYN levels were decreased in endogenous anxiety and normalized after treatment [144]. Metabolomic studies reported decreased KYN levels in patients diagnosed with Type D personality that is characterized by negative affectivity and social inhibition [145]. Stress and inflammation appear to activate the TRP-KYN pathway, depleting 5-HT and melatonin and thus making more susceptible to anxiety (Table 2).

### 3.4. Substance Use Disorder

Substance use disorder (SUD) affects a person’s brain and behavior leading to an inability to control the use of a drug or medication. In addition to an impaired control, patients show social impairment, risky use and pharmacological indicators including tolerance and withdrawal. Common substances are alcohol, sedatives, caffeine, hallucinogens, inhalants, stimulants and tabaco, among others [146]. SUD is frequently comorbid with other mental illnesses. Positive symptoms are more prominent among substance abusing SCZ patients [147]. The onset of positive symptoms occurs in nearly three-fourths of cannabis users after cannabis abuse [148]. Serious hallucinations and delusions are frequent in alcohol addicted SCZ patients [149]. Negative symptoms are less common in patients with substance use disorder probably because patients with social withdrawal have more difficulties to obtain abused substance [150]. Increased rates of criminal activity and violent behavior are more common in SCZ patients with SUD [151]. Cognitive impairments are prevalent among patients with SUD. Alcohol affects total and memory domain scores more than cannabis, while opioids affect visuospatial domain more than cannabis or stimulants [152]. SUD occurs at an increasing rate in patients with GAD. Substance use and anxiety are considered to occur in a vicious cycle [153] (Table 2).

Few studies regarding inflammatory cytokines were reported. Cocaine increased the mRNA expression of IL-1ß receptor in the ventral tegmental area, reducing cocaine seeking. It may suggest that chronic cocaine use induces proinflammatory signaling contributing to cocaine seeking [154]. A single nucleotide polymorphism in the IL-10 gene is associated with decrease expression of IL-10 and is linked to alcoholism. It was suggested that increased proinflammatory and reduced anti-inflammatory signals are predisposing factors for alcoholism [155] (Table 2). 

No clinical study was found regarding the serum or CSF level of KYNs in patients with SUD. The TRP-KYN metabolic pathway is considered to play an important role in SUD and was discussed as a potential target for SUD therapy [17]. Patients with cocaine use disorder (CUD) frequently develop MDD. The plasma 5-hydroxytryptamine (5-HT) concentration was significantly higher and the KYN/5-HT ratio was significantly lower in patients with CUD-induced MDD than those with MDD, while there were no differences between CUD-primary MDD and MDD. It suggests that the TRP-KYN pathway participates less in CUD-induced MDD and the presence of other mechanisms of the development of depression [156] (Table 2).

### 3.5. Post-Traumatic Stress Disorder

Post-traumatic stress disorder (PTSD) develops after a terrifying experience in individuals who suffer from flashbacks, nightmares, severe anxiety and uncontrollable thoughts regarding the event. Substantial evidence supports that PTSD is caused by insufficient integration of a trauma memory into the hippocampal-cortical memory networks, forming fragmented, incomplete and disorganized intrusive memories [157]. Most patients with PTSD complained of chronic sleep disturbances characterized by significantly reduced slow wave sleep (SWS), light sleep stages, awakenings, arousals and increased rapid eye movement (EEG). SWS drives memory consolidation by repeated reactivation of newly encoded memory. Thus, lowered sleep quality facilitates the formation of intrusive memories [158]. PTSD is frequently comorbid with SCZ. More than a half of patients with PTSD experienced psychotic positive symptoms, but emerging evidence suggests that PTSD with secondary psychosis might be different from PTSD without psychosis [159]. Patients with PTSD activate the fight-flight-freeze response and reduce overall brain functioning, leading to several negative symptoms [160]. PTSD causes long-term cognitive dysfunction such as memory, attention, planning and problem solving [161]. PTSD frequently cooccur with GAD and their symptoms overlap [162] (Table 2).

Inflammation has been linked to PTSD. Increased proinflammatory cytokines IL-1β, IL-6, IFN-γ and TNF-α were found elevated in the serum of patients with PTSD and partly correlated with the severity of PTSD [163]. Decreased levels of anti-inflammatory cytokine IL-4 were reported in patients with PTSD. The alteration of the serum anti-inflammatory cytokines IL-4 and IL-10 remains inconclusive in PTSD [164]. Higher levels of serum anti-inflammatory cytokine TGF-β were found to be predicative indicators for the development of PTSD one month after accidents [165] (Table 2).

No clinical study was reported regarding the peripheral or CSF samples of KYNs in patients with PTSD. KYN metabolites are monitored in clinical settings as evidence of inflammatory responses contributing to sleep deprivation and the formation of intrusive memories [164].

### 3.6. Schizophrenia

SCZ is a mental disorder in which patients abnormally interpret reality and suffer from hallucinations, delusions and extremely disordered thinking and behavior. Patients with SCZ usually experience positive symptoms such as hallucinations, delusions, flight of ideas, negative symptoms such as apathy, emotionless, lack of social functioning and cognitive symptoms including difficulty in concentration and attention, and memory impairments. However, cognitive symptoms are subtle and are often detected only when neuropsychological tests are performed [166]. The prevalence of GAD was significantly higher in patients with SCZ and the prevalence of panic disorder, social anxiety disorder and obsessive-compulsive disorder was significantly higher in SCZ patients [167] (Table 2).

The peripheral activation of the immune system was observed in SCZ. Three meta-analyses on serum cytokines of SCZ patients were reported accordingly. (1) IL-1β, IL-6 and TGF-β were increased in acutely relapsed and first-episode psychosis and the cytokine levels were normalized with antipsychotic treatment. Soluble IL-2 receptor (sIL-2R) stayed high in acute psychosis and after antipsychotic treatment [168]. (2) IL-6, TNF-α, sIL-2R and IL-1 receptor antagonist were significantly increased in acutely exacerbating and IL-6 levels significantly decreased following treatment. IL-1β and sIL-2R were significantly increased in chronic SCZ [123]. (3) MCP-1 (CCL2), MIP-1β (CCL4), eotaxin-1 (CCL11) and IL-8 were elevated in pooled analysis of all SCZ patients, while MCP-1 was elevated in first-episode psychosis (FEP) and IL-8, eotaxin-1 and MIP-1β were elevated in multiple-episode psychosis [169] (Table 2).

The activation of the central immune system was also observed. Three meta-analysis on CSF cytokines of SCZ were reported accordingly. (1) IL-1β was decreased significantly decreased in SCZ, but there was no significant difference in CSF levels of IL-1α, IL-2 or IL-6 between SCZ and healthy controls [168]. (2) IL-1β, IL-6 and IL-8 were significantly increased IL-2R were significantly decreased in SCZ [125]. (3) IL-6 and IL-8 were significantly elevated in SCZ and IL-6 levels were higher in early-stage SCZ than chronic SCZ [170] (Table 2). CRP, IL-6 and TNF-α are overlapping biomarkers in SCZ and cardiovascular diseases and anti-inflammatory drugs were proposed for the treatment of SCZ [171].

Regarding the KYN system, the serum KYN and KYN/TRP ratio was higher in SCZ [172]. A meta-analysis of CSF samples showed increased KYN and KYNA levels and another meta-analysis of plasma, CSF, brain tissue or saliva showed increased levels of KYNA in SCZ [125,173] (Table 2). Thus, the KYN system is activated in SCZ and elevated KYNA levels are considered to contribute to the cognitive impairments of SCZ. Recently, another meta-analysis reported that KYNA levels and the KYNA/3-HK ratio were not altered and the KYNA/KYN ratio was decreased in SCZ, suggesting the presence of differential pattern between SCZ and mood disorders [174]. The combination of acetylcholine inhibitor galantamine and N-methyl-D-aspartate receptor memantine was proposed as antioxidant treatment SCZ and for the treatment of SCZ cognitive impairments [175,176].

### 3.7. Autism Spectrum Disorder

Autism Spectrum Disorder (ASD), defined by the Diagnostic and Treatment Manual for Mental Disorders, Fifth Edition (DSM-5), is characterized by persistent deficits in social communication interaction and restricted-repetitive patterns of behavior, interests or activities. A specific subtype of ASD is linked to comorbid psychosis, showing positive symptoms of delusion, hallucination, thought disorder, mania and depression and negative symptoms of psychosis appear to share many features with ASD [177]. Cognitive deficits including mental deterioration, are associated with social and communication difficulties that involve components of cognition, communication and social understanding [178]. Meta-analyses reported that ASD children had higher anxiety levels than normally developing ones, that high-functioning ASD adolescents are at high risk of developing anxiety disorders, and that autism population showed higher prevalence of anxiety disorders, the highest being attention-deficit hyperactivity disorders, in decreasing orders, sleep-wake disorders, disruptive, impulse-control and conduct disorders depressive disorders, obsessive-compulsive disorder BD and SCZ spectrum disorders [179,180] (Table 2).

Regarding inflammatory cytokines, two Meta-analyses showed increases of IL-1β, IL-6, IL-8, IFN-γ, TNF-α, eotaxin and monocyte chemotactic protein-1, while TGF-β1 were significantly lower in ASD [181,182]. However, a case-control study reported that significantly higher levels of IL-4, IL-5 and IL-13, with Th-2 predominance in plasma and peripheral blood mononuclear cells of ASD children [183] (Table 2).

The alteration of the KYN system was also observed. The mean serum level of KYNA was significantly lower, while the KYN/KYNA ratio was significantly higher in children with ASD. The same relative values were found when comparing the childhood autism subgroup with the controls [184]. Significantly higher KYN/TRP ratio and KYN and QUIN levels were observed in blood samples of ASD patients, while no significant difference of KYNA and significantly lower picolinic acid level were detected in ASD [185] (Table 2).

All psychiatric disorders presented an evidence of the innate inflammatory activation by increased pro-inflammatory cytokines. MDD, BD, SCZ and ASD witnessed the activation of the secondary adaptive immune response by increased anti-inflammatory cytokines, while GAD, and SUD showed reduced levels of anti-inflammatory cytokines. PTSD showed no changes or mixed results depending on the cause of stress. Regardless of the activation of the secondary adaptive immune response, the inflammatory profiles of all psychiatric disorders described in this review, have shifted away from healthy state (Table 2).

Either causative or resultant of acute and chronic inflammation, the altered balance of toxic and protective KYN metabolites was observed. Toxic KYN metabolites are increased in MDD, GAD, SCZ, ASD and the CSF samples of BD. Modulatory KYN metabolites were increased in SCZ; decreased in MDD and ASD; unchanged in the serum of BP; unknown in GAD and SUD.

## 4. Conclusions and Future Perspective

LGI defined by the serum concentration of CRP has well established the relationship with physical health score in healthy individual, but not mental health score. Thus, the CRP measurement is not adequate for the assessment of mental health. LGI is also characterized by a long period of slightly elevated serum concentrations of proinflammatory and anti-inflammatory factors and accompanying immune tolerance. Elevated levels of proinflammatory cytokines are consistent findings in major psychiatric disorders, but the levels of anti-inflammatory cytokines are mixed, and their roles in the pathogenesis remain obscure. Some inflammatory cytokines and factors cannot be simply categorized into pro- or anti-inflammatory; thus, comprehensive analysis according to their specific functions in immune reaction may further complement this study. 

The involvement of toxic KYNs in mental disorders has gained credit and this study reinforced current understanding of their interactions with the immune system in LGI. However, the roles of toxic KYNs in BP, SUD and PTSD are to be explored. The roles of protective KYNs such as KYNA, AA, PA and CA are even more obscure or unknown. The decreased levels of KYNA are considered to contribute to the pathogenesis of MDD and ASD, while the increased level of KYNA is considered to be at least one of the culprits in the exacerbation of SCZ.

Generally, the measurement of TRP/KYN ratio is used for assessment of the activation of the TRP-KYN pathway and 3-HK/KYNA and QUIN/KYNA ratios are used for relative toxicity of the KYN system. However, emerging evidence has suggested KYN metabolites cannot be simply categorized into toxic or protective. For example, KYNA is excitatory in low dose at α-amino-3-hydroxy-5-methyl-4-isoxazolepropionic acid (AMPA) receptor, while inhibitory in high dose at AMPA receptor. Our preliminary data indicated that KYNA elicits cognitive enhancement in a low dose, but not in higher doses. 3-HK is generally considered to be oxidative and thus toxic, but it may serve as antioxidant in particular environment. Metabolomic analysis of KYN metabolites and their related molecules may help identify a certain profile contributing to LGI and its exacerbation. Furthermore, the influence of nutritional, metabolic and sleep status on the structure and the function of the brain is of particular interest [186,187,188].

The immune privileged site of CNS encapsulated by the BBB may complicate the assessment of inflammatory status in the brain. The BBB is semipermeable; thus, the entry of circulating molecules into the CNS depends on the state of the BBB. The integrity measurements of the BBB may ensure the assessment of LGI in the CNS and functional imaging studies may reveal a relationship with psychiatric symptoms in mental illnesses [189]. Together, they are expected to warrant as additional indicators for a battery of biomarkers which may serve as risk, diagnostic, prognostic, predicative and/or therapeutic biomarker in psychiatric diagnosis.

Finally, the classification of psychiatric disorders mainly depends on the manifestation of signs and symptoms, which inevitably includes a broad range of heterogenous population into study samples, leading to inconclusive results. Frequent comorbidities of mental illnesses even more complicate the procedures of grouping subjects. Application of research domain criteria and artificial intelligence diagnostic system which integrate many levels of information may become a useful assist in focusing on homogenous populations with the reduction of statistical deviations [190].

Understanding the roles of chronic LGI in the development of mental illnesses, the TRP-KYN metabolic pathway in chronic LGI including mechanism of the immune switch, dynamic structural changes of the BBB and the participation of components of the body in neuroinflammation will certainly help explore possible preventive, interventional and therapeutic measures through the TRP-KYN pathway.

## Figures and Tables

**Table 1:** The enzymes of the tryptophan-kynurenine pathway and related diseases.

Enzymes	Substrates	Products	Diseases
TDO	Tryptophan	L-kynurenine	Human brain tumors [34] Other tumor types [35,36]
IDO	Tryptophan	L-kynurenine	Tumors [36,37] Neurological and neurodegenerative diseases [25] Depression [38] Systemic lupus erythematosus [33] Sjörgen’s syndrome [31,32]
KAT	L-kynurenine 3-hydroxy-L-kynurenine	Kynurenic acid Xanthurenic acid	Multiple sclerosis [39] Parkinson’s disease [40] Alzheimer’s disease [41] Huntington’s disease [42,43,44] Epilepsy [25] Amyotrophic lateral sclerosis [45] Schizophrenia [46,47,48]
KMO	L-kynurenine	3-hydroxy-L-kynurenine	Bipolar disorder [49] Autoimmunity related diseases [50]

**Table 2:** The enzymes of the tryptophan-kynurenine pathways and related disease.

		Major Depressive Disorder	Bipolar Disorder	Generalized Anxiety Disorder	Substance Use Disorder	Post-Traumatic Stress Disorder	Schizophrenia	Autism Spectrum Disorder
Symptoms	Positive	+	++	++	++	++	++	+
	Negative	++	++	++	+	++	++	+
	Cognitive	+	+	++	++	++	-	+
	Anxiety	++	++	++	++	++	++	++
Inflammatory factors	Proinflammatory	↑	↑	↑	↑	↑	↑	↑
	Anti-inflammatory	↑	↑	↓	↓	↓,?	↑	↑
Kynurenines	Toxic	↑	?	↑	?	?	↑	↑
	Protective	↓	? (serum), ↑ (CSF)	?	?	?	↑,?	↓

+: noticeable; ++: prominent; -: not typical;↑: increase; **↓**: decrease; ?: questionable or unknown.

## Data Availability

Not applicable.

## Abbreviations

AA	anthranilic acid
AhR	aryl hydrocarbon receptor
AMPA	α-amino-3-hydroxy-5-methyl-4-isoxazolepropionic acid
APC	antigen-presenting cell
ASD	autism spectrum disorder
ATP	adenosine triphosphate
BBB	blood-brain barrier
BD	bipolar disorder
CA	cinnabarinic acid
CCBL	cysteine conjugate beta-lyase
CNS	central nervous system
CRP	C-reactive protein
CSF	cerebrospinal fluid
CUD	cocaine use disorder
DSM-5	Diagnostic and Treatment Manual for Mental Disorders, Fifth Edition
EEG	electroencephalography
FEP	first-episode psychosis
GAD	generalized anxiety disorder
GPR35	G-protein coupled receptor 35
3-HAA	3-hydroxyanthranilic acid
3-HK	3-hydroxy-kynurenine
HD	Huntington’s disease
HRQL	Health-Related Quality of Life
5-HT	serotonin, 5-hydroxytryptamine
IDO	indoleamine 2,3-dioxygenase
IFN	interferon
IL	interleukin
iNKT	invariant natural killer cell
KATs	kynurenine aminostransferase
KMO	kynurenine 3-monooxygenase
KP	kynurenine pathway
KYN	kynurenine
KYNA	kynurenic acid
LGI	low-grade inflammation
MCP	monocyte chemoattractant protein
MDD	major depressive disorder
MS	multiple sclerosis
MT	melatonin
NAD^+^	nicotinamide adenine dinucleotide
NADPH	nicotinamide adenosine dinucleotide phosphate
NK	natural killer cell
PD	Parkinson’s disease
PIC	picolinic acid
PTSD	post-traumatic stress disorder
QUIN	quinolinic acid
ROS	reactive oxygen species
SANS	Scale for the Assessment of Negative Symptoms
SCZ	schizophrenia
sIL-2R	soluble IL-2 receptor
SjS	Sjögren’s syndrome
SLE	systemic lupus erythematosus
SUD	substance use disorder
SWS	slow wave sleep
TDO	tryptophan 2,3-dioxygenase
Th1	T helper type 1
Th17	T helper type 17
Th2	T helper type 2
TNF	tumor necrosis factor
TRP	tryptophan
XA	xanthurenic acid
